# Linagliptin ameliorates tacrolimus-induced renal injury: role of Nrf2/HO-1 and HIF-1α/CTGF/PAI-1

**DOI:** 10.1007/s11033-024-09533-2

**Published:** 2024-05-05

**Authors:** Mohamed E. Nady, Ola M. Abd El-Raouf, El-Sayed M. El-Sayed

**Affiliations:** 1 Department of Pharmacology & Toxicology, Faculty of Pharmacy, Al-Azhar University, Cairo, Egypt; 2 Pharmacology Department, Egyptian Drug Authority (EDA), formerly known as National Organization for Drug Control and Research (NODCAR), 6 Abou Hazem St., Pyramids Ave, Giza, Egypt

**Keywords:** Oxidative stress, Hypoxia, Apoptosis, Renal fibrosis, DPP4 inhibitors

## Abstract

**Background:**

Tacrolimus (TAC) is a frequently used immunosuppressive medication in organ transplantation. However, its nephrotoxic impact limits its long-term usage. This study aims to investigate the effect of linagliptin (Lina) on TAC-induced renal injury and its underlying mechanisms.

**Methods and results:**

Thirty-two Sprague Dawley rats were treated with TAC (1.5 mg/kg/day, subcutaneously) and/or Lina (5 mg/kg/day, orally) for 4 weeks. Histological examination was conducted, and serum and urinary biomarkers were measured to assess kidney function and integrity. Furthermore, ELISA, Western blot analysis and immunohistochemical assay were employed to determine signaling molecules of oxidative stress, profibrogenic, hypoxic, and apoptotic proteins. Tacrolimus caused renal dysfunction and histological deterioration evidenced by increased serum creatinine, blood urea nitrogen (BUN), urinary cystatin C, and decreased serum albumin as well as elevated tubular injury and interstitial fibrosis scores. Additionally, TAC significantly increased the expression of collagen type-1, alpha-smooth muscle actin (α-SMA), plasminogen activator inhibitor-1 (PAI-1), and transforming growth factor-beta1 (TGF-β1) renal content. Moreover, TAC decreased the expression of nuclear factor erythroid-2-related factor2 (Nrf2), heme oxygenase 1 (HO-1), and mitochondrial superoxide dismutase (SOD2). In addition, TAC increased protein expression of hypoxia-inducible factor1-alpha (HIF-1α), connective tissue growth factor (CTGF), inducible nitric oxide synthase (iNOS), 8-hydroxy-2-deoxyguanosine (8-OHdG), as well as nitric oxide (NO), 4-hydroxynonenal, caspase-3 and Bax renal contents. Furthermore, TAC decreased Bcl-2 renal contents. The Lina administration markedly attenuated these alterations.

**Conclusion:**

Lina ameliorated TAC-induced kidney injury through modulation of oxidative stress, hypoxia, and apoptosis related proteins.

**Supplementary Information:**

The online version contains supplementary material available at 10.1007/s11033-024-09533-2.

## Introduction

Tacrolimus is the most frequently utilized immunosuppressive calcineurin inhibitor in solid organ transplantation; it increases grafted organ survival and is used as a treatment option for various autoimmune disorders [1]. However, prolonged use causes nephrotoxicity [2]. Tacrolimus causes arteriolar vasoconstriction that results in decreased renal blood flow and glomerular filtration; and the consequent hemodynamic alterations cause ischemia, tubular dysfunction, and apoptosis which ultimately promote tissue fibrosis and kidney damage [3, 4].

The pathogenesis of the nephrotoxic effect of tacrolimus remains unclear. However, oxidative stress is suggestive as a common mechanism that has been reported in many studies [5–7]. In addition, hypoxia has been hypothesized as a major contributor to TAC-induced renal injury that results in tissue deterioration, and fibrosis [8]. HIF-1α is a crucial nuclear transcription factor involved in maintaining O_2_ homeostasis, and cellular adaptation to hypoxia. It is evident that HIF-1α may promote extracellular matrix remodeling to mediate renal fibrosis by inducing inflammation, epithelial-mesenchymal transition, and collagen deposition [9]. It was also reported that HIF-1α upregulates various pro-fibrogenic factors, such as CTGF, and PAI-1, during the progression of kidney injury [10].

Dipeptidyl peptidase-4 (DPP-4) is a multifunctional aminopeptidase protein that is highly expressed in kidney endothelial, and proximal tubular cells [11]. Linagliptin is a selective and potent xanthine-based DPP-4 inhibitor. The protective effects of Lina on renal impairment were clinically proven and extensively studied in different experimental models of acute and chronic kidney diseases [12, 13]. Collectively, these effects were attributed to its antioxidant and free radical scavenging activity, anti-inflammatory, and anti-fibrotic properties. In addition, a recent report showed that Lina abrogates hypoxia-induced neuronal damage, and potentiates Nrf-2/ HO-1 signaling in a non-diabetic rat model of Parkinson’s disease [14].

To the best of our knowledge, no previous work has described the protective effects of Lina against TAC nephrotoxicity. Therefore, this study aims to evaluate the impact of the antidiabetic DPP-4 inhibitor, Lina on TAC-induced kidney injury beyond the improvement of glycemic control and emphasize the modulatory effects on Nrf2/HO-1 and HIF-1α/CTGF/PAI-1 axis.

## Materials and methods

### Chemicals

Tacrolimus (Prograf®) was purchased from Astellas Toyama Co. Ltd. (Japan); and linagliptin was provided by Honour Lab Limited (Telangana, India). Any other chemicals or reagents were of analytical grade, and obtained from local commercial sources. The designated dose of Lina was suspended in distilled water while TAC was diluted in olive oil to a final concentration of 1 mg/ml.

### Animal care

Thirty-two adult male Sprague Dawley rats approximately weighing 200–220 g were obtained from the Laboratory Animal Colony of Helwan (Cairo, Egypt). The animals were housed for two weeks for acclimatization with regular 12/12 h light/dark cycles, in a constant room temperature of 25 ± 2 °C, and a relative humidity of 55 ± 5%. They were also kept with free access to tap water *ad libitum*, and low salt diet pellets (Raw Bright International Company, Shebin EL Kom, Menoufia, Egypt). A low-salt diet was used to increase TAC nephropathy [15].

### Study design and treatment protocol

Thirty-two adult rats were randomly and equally allocated into 4 groups (*n* = 8/group), and treated once daily for 4 weeks as follows:

- **Group 1**(Control): Rats received olive oil (1 ml/ kg/day, subcutaneously).
- **Group 2** (Lina): Rats received linagliptin (5 mg/kg/day, p.o.).
- **Group 3** (TAC): Rats received tacrolimus (1.5 mg/kg/day, subcutaneously).
- **Group 4** (TAC + Lina): Rats received TAC (1.5 mg/kg/day, subcutaneously) followed by Lina (5 mg/kg/day, p.o.). The appropriate dosing and duration of treatment drugs were chosen according to the previous studies [16, 17], and were additionally confirmed based on our primary pilot study (data not shown).

### Sampling collections

To obtain urine samples, the animals were housed individually in metabolic cages just 24 h before euthanasia. Then, urines were collected, and centrifuged at 5000 x g for 10 min to remove urine debris. After that, they were kept frozen at -20 °C until used for further investigations.

Under mild ether anesthesia, sufficient volumes of blood were withdrawn from the retro-orbital venous plexus of each rat, and put into non-heparinized micro-capillaries [18]. The sera were separated using a cooling centrifuge (Z446-K, Hermle Labortechnik, Germany) at 3000 rpm for 15 min at 4 °C. Next, they were stored at -20 °C for biochemical analysis. Subsequently, the rats were euthanized by cervical dislocation technique according to the standard animal euthanasia method guidelines developed by the Canadian Council on Animal Care. After that, the rats’ kidneys were collected, weighed, longitudinally sectioned, and divided into parts. A portion was fixed in a 10% neutral buffered formalin solution and processed for immunohistochemical and histological evaluation. Finally, the remainder were kept at -80° C for total protein determination, ELISA assay, and Western blot analysis.

### Assessment of kidney function biomarkers

Urinary and serum creatinine (S_cr_) were measured using a quantitative kinetic method assay kit obtained from a local supplier (Biodiagnostic, Catalog No. CR2050, Giza, Egypt). In addition, BUN (Catalog No. 318-001) and serum albumin (Catalog No. 211-001) were quantified colorimetrically according to the standard manufacturer’s instructions (Spectrum Diagnostics, Cairo, Egypt). BUN/ S_cr_ ratio as well as creatinine clearance (Cl_Cr_) were calculated as previously described [19, 20]. Additionally, urinary cystatin C was estimated using an ELISA assay Kit (MyBioSource Inc., Catalog No. MBS704068, CA, USA), according to the supplier’s directions.

### Determination of nitric oxide content in kidney tissues

Nitric oxide was evaluated by measuring the level of nitrite, a by-product of NO in the kidney tissue homogenate using Griess reagent, as described by Miranda et al. [21], according to manufacturer’s directions (Biodiagnostic, Catalog No. NO25-33, Giza, Egypt).

### Histopathological examination

The formalin-fixed and paraffin-embedded (FFPE) kidney sections were cleared in xylene, and rehydrated in ascending grades of ethyl alcohol, and 5 μm tissue sections were stained with either hematoxylin & eosin (HE) to examine the renal injury, or Masson’s trichrome to assess the level of collagen deposition, according to standard methods of assay [22].

A semi-quantitative evaluation was performed at six non-overlapping fields that were randomly selected from cortical regions under the light microscope (Olympus IX71, Japan).

The captured photographs were used to investigate the severity of histopathological changes in kidney tissues by a pathologist blinded to the identity of the treatment protocol. The microscopic alterations have received the following score: (1) normal histology, alterations affect less than 25% of the cross-section; (2) changes affect 25–50% of the section; (3) changes affect 50–75% of the section; and (4) changes affect more than 75% of the cross-section. Moreover, for Masson’s staining, at least ten random non-overlapping fields were selected under the light microscope at 400x magnification (Leica DM500, Leica Biosystems, Germany). Tubulointerstitial fibrosis was distinct by a matrix-rich expansion of the interstitium shown in blue color. Furthermore, the degree of collagen deposition in each group was measured using a computed image analyzer (Leica LMD Software, Germany), and expressed as a relative ratio of the total cortical area.

### ELISA assays

The renal content of TGF-β1 was estimated using a rat transforming growth factor β1 ELISA kit obtained from Cusabio® (Catalog No. CSB-E04727r, Houston, Texas, USA). Moreover, the renal caspase-3 content was determined by a rat caspase-3 ELISA kit provided by Kamiya Biomedical Company (Catalog No. KT 9429; Seattle, WA, USA). In addition, the apoptotic marker, Bax (Catalog No. MBS2512405), and the anti-apoptotic marker, Bcl2 (Catalog No. MBS2515143) as well as the kidney tissue content of 4-hydroxynonenal (Catalog No. MBS3808906) were determined using rat ELISA Kits, according to manufacturer’s instructions (MyBioSource Inc., San Diego, CA, USA).

### Immunohistochemical investigation

The immunohistochemical assay method was performed according to Abd El-Lateef et al. [23]. The heat-induced epitope retrieval technique was applied to FFPE tissue sections at 95 °C for 5 min. kidney sections were incubated with the primary antibodies obtained from Santa Cruz Biotechnology Inc. (Heidelberg, Germany) including COL1α1(sc-293,182), PAI1 (sc-5297), Nrf2(sc-365,949), HO-1(sc-390,991), SOD2(sc-137,254), iNOS (sc-7271), and 8-OHdG (sc-66,036) along with α-SMA (Dako IR611) that purchased from Agilent Technologies Inc. (Santa Clara, CA, USA) at dilutions of 1:100 for 1 h at 37 °C. After washing, the sections were incubated with HRP-conjugated goat anti-rat IgG secondary antibody obtained from Thermo Fisher Scientific (Cat. No. 31,470; MA, USA) at a dilution of 1:10000. Then, the tissue sections were auto-stained with a high-sensitivity visualization system (Envision™ FLEX, High pH, and Link system, Agilent Technologies, USA) for 30 min at 37 °C. The sectioned slides were examined using a light microscope to assess the expression of tested antibodies in all treated groups. At least ten images from non-overlapping fields were captured at 400x magnification. Eventually, the immunoreactivity was assessed by estimating the area percentage of positively immunostained cells utilizing ImageJ software (Madison, WI, USA), and the color deconvolution plugin.

### Western blot analysis

The Western blotting technique was performed as previously described [3]. Briefly, kidney tissue was homogenized, and total protein was estimated using the Bradford method [24]. Then twenty µg of protein from each homogenate was loaded onto gradient polyacrylamide gels (4–12%; Bio-Rad Laboratories), and transferred to nitrocellulose membranes. Next, the membranes were blocked in 3% bovine serum albumin for 1 h, and then probed overnight at 4 °C with rat monoclonal antibodies obtained from Santa Cruz Biotechnology Inc. (Heidelberg, Germany) including HIF-1α (Catalog No. sc-13,515), and CTGF (Catalog No. sc-101,586) at dilutions of 1:1000, and β-actin (Catalog No. sc-47,778, 1:5000). Next day, the membranes were washed using tris-buffered saline with 0.1% tween 20 for 5 min. After that, the membranes were incubated with horseradish peroxidase conjugated to goat anti-rat-IgG-AP secondary antibody (Cat. No. A18868, Thermo Fisher Scientific, MA, USA) against the blotted target protein for 1 h at dilution of 1:5000 at room temperature. The signals were detected using an enhanced chemiluminescence detection reagent (Bio-Rad Laboratories, Catalog No. 170–5060, CA, USA). The signal intensity was analyzed using ChemiDoc MP Imager (Bio-Rad Laboratories, CA, USA) and quantified using the Image Lab Software (Bio-Rad Laboratories, CA, USA). The protein expression levels were normalized to β-actin.

### Statistical analysis

The results are expressed as means ± standard deviations (SD). All analyses were performed using GraphPad Prism software version 8.0.2 (San Diego, CA, USA). Differences among groups were statistically analyzed by one-way analysis of variance (ANOVA), followed by the Tukey-Kramer post-hoc test for multiple comparisons. The non-parametric data for tubular injury score were analyzed by the Kruskal-Wallis test followed by Dunn’s post-hoc test. Statistical significance was set at *P* < 0.05.

## Results

### Effect of Lina administration on kidney function indicators

**Fig. 1:**
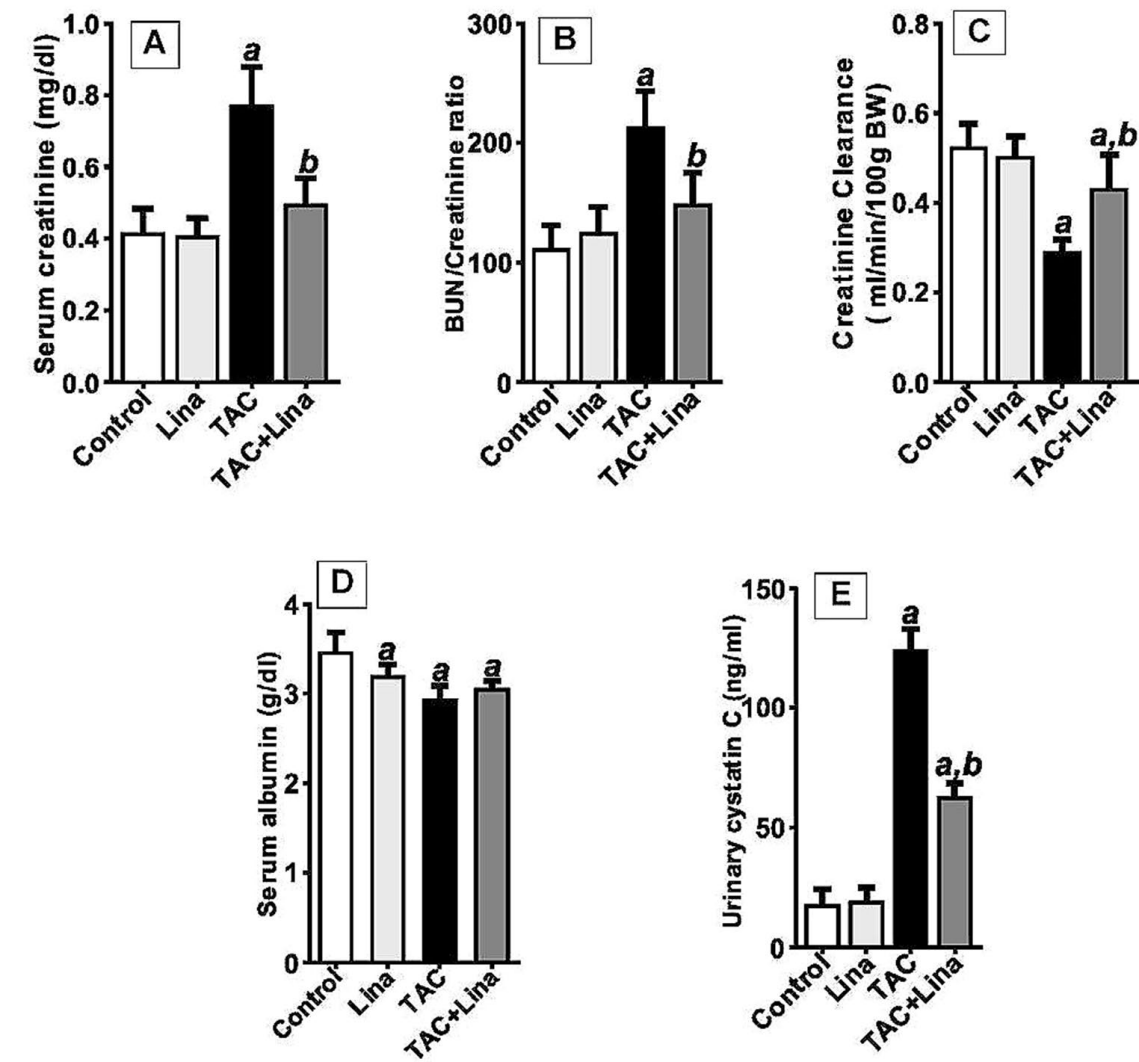
Effect of Lina treatment on kidney function biomarkers in TAC-induced renal injury in rats. (**A**)Serum creatinine (mg/dl), (**B**) BUN/Creatinine ratio, (**C**) Creatinine Clearance (ml/min/100 g BW), (**D**) Serum albumin (g/dl), (**E**) ELISA assay of urinary cystatin C (ng/ml). Data are presented as means ± SD (*n* = 6 per group) and statistically analyzed by a one-way ANOVA test, followed by a post hoc Tukey-Kramer multiple comparison test. ***a***: significantly different from control group, ***b***: significantly different from TAC group at *P* < 0.05

Data analysis of the kidney injury biomarkers (S_cr_, BUN/S_cr_ ratio, and urinary cystatin C) indicated a statistically significant increase in the levels of these biomarkers in the TAC-treated group amounting to 83%, 85%, and 591%, respectively, compared to the control group. Additionally, the serum albumin, and Cl_Cr_ showed a statistically significant reduction to 17%, and 44%, respectively. Conversely, Lina co-treatment remarkably decreased the levels of S_cr_, BUN/S_cr_ ratio, and urinary cystatin C to 35%, 28%, and 48.7%, respectively. In addition, the co-treatment group manifested a higher level of Cl_Cr_ amounting to 48%, compared to the TAC group (Fig. 1).

### Effect of Lina on histopathological changes induced by TAC

**Fig. 2:**
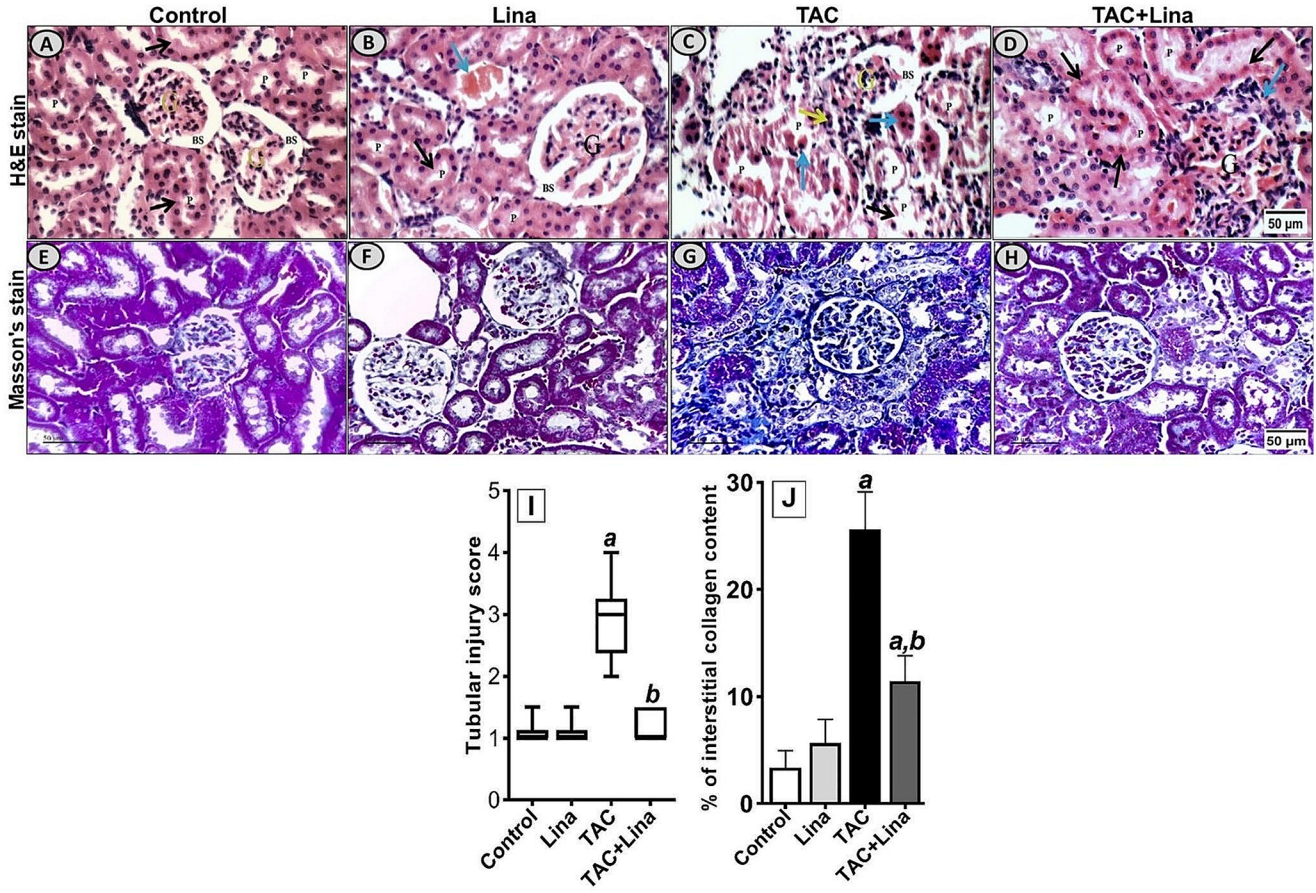
Histopathological examination of kidney tissue sections collected from different treatment groups of male Sprague Dawley rats. (**A**-**D**): Representative micrographs for kidney tissue section stained with H& E; (**I**): Representative semi-quantitative tubular injury score (*n* = 6 per group). The images were captured under a light microscope at 400x magnification. The scale bar represents 50 μm. **(A; Control)**: kidney showing average glomeruli (**G**), with average Bowman’s spaces (BS), and proximal tubules (P) with preserved brush borders; **(B; Lina)**: kidney showing average G with average BS, average P with preserved brush borders (black arrow), and mildly dilated congested interstitial blood vessel (blue arrow); **(C; TAC)**: kidney showing atrophied glomerulus with widened BS, proximal tubules with complete loss of brush borders (black arrows) and markedly apoptotic epithelial lining (blue arrow), and marked interstitial inflammatory infiltrate (yellow arrow); **(D; TAC + Lina)**: kidney showing proximal tubules with apoptotic epithelial lining (black arrow) and mild interstitial inflammatory infiltrate (blue arrow). Data are expressed as mean ± SD. Statistical significance was determined by the Kruskal-Wallis test (non-parametric one-way ANOVA), followed by Dunn’s post-hoc test for the multiple comparisons between the groups at *P* < 0.05. (**E-H**): Representative photographs of kidney tissue sections stained with Masson’s trichrome captured under a Leica microscope at 400x magnification, scale bar represents 50 μm. (**J**) represents the quantitative score of tubulointerstitial collagen content in the renal cortex (*n* = 10 per group). The data are represented as mean ± SD. Statistical significance was determined by one-way ANOVA followed by theTukey-Kramer test for multiple comparison at *P* < 0.05. **a**: significantly different from the control group, **b**: significantly different from TAC group

Microscopical analysis of H&E stained kidney sections revealed obvious tissue deterioration as well as a statistically significant increase in tubular injury score in the TAC group. This is in comparison to the control group (Fig. 2A), where renal sections from TAC-treated rats (Fig. 2C) showed glomerular, tubular, and interstitial injury comprising atrophied glomeruli with widened Bowman’s spaces, proximal tubules with complete loss of brush borders, marked apoptotic epithelial lining, marked interstitial inflammatory infiltrate, and striped fibrous lesions. On the contrary, these pathological alterations were improved by Lina co-treatment (Fig. 2D). In addition, Lina administration significantly decreased the tubular injury score (Fig. 2I), when compared to the TAC group. The Masson’s trichrome stain was used to evaluate the effect of Lina treatment on the tubulointerstitial fibrosis caused by TAC (Fig. 2E-H). Our results have indicated a significant increase in collagen deposition in the tubulointerstitial region following TAC treatment, as evidenced by an increase in blue staining (733%), compared to the control group. In contrast, the tissue sections from the Lina co-treated group showed a significant decrease in extracellular matrix expansion by 55%, compared to the TAC group (Fig. 2J).

### Effect of Lina on the profibrotic molecules induced by TAC

**Fig. 3:**
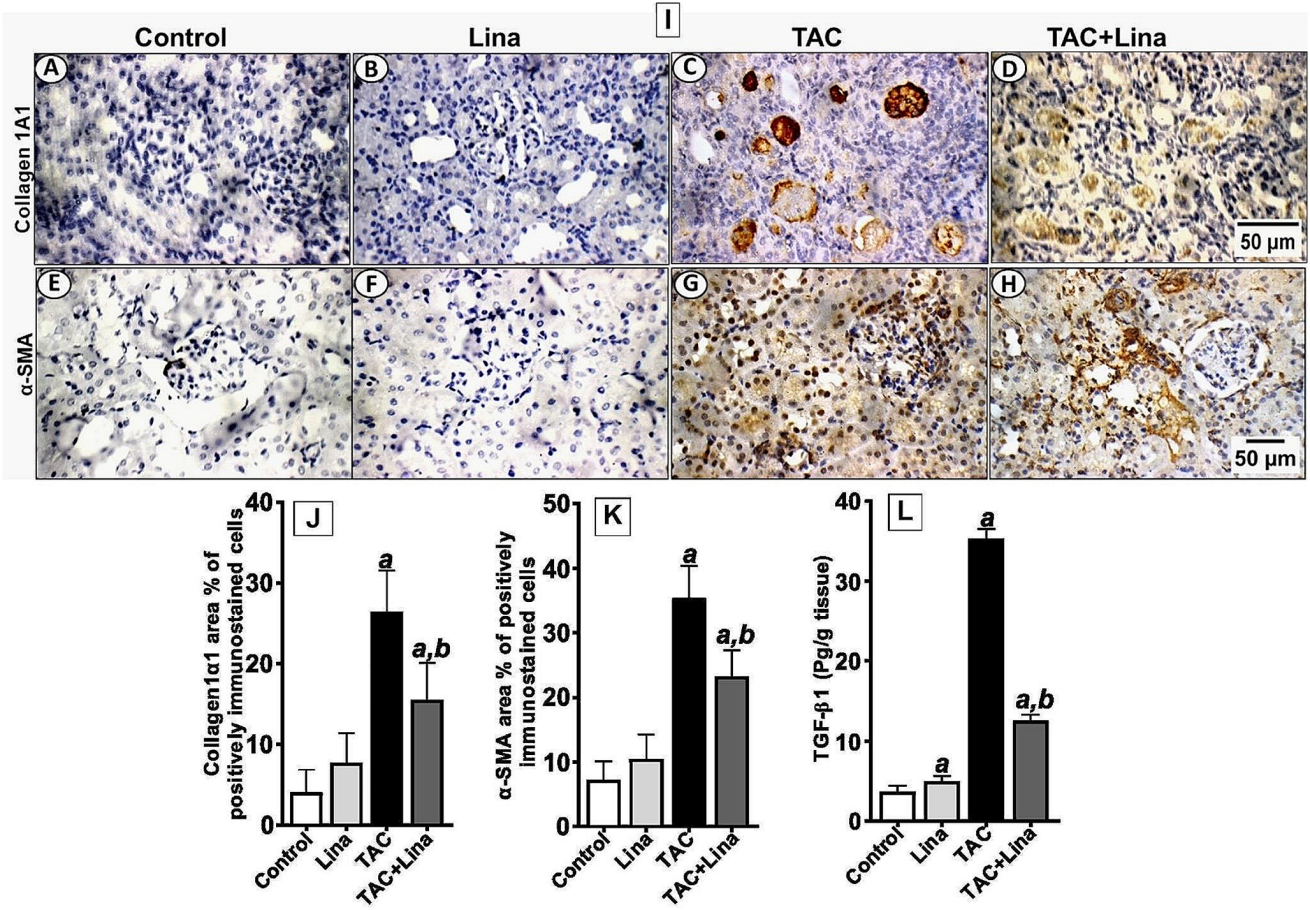
Effect of Lina treatment on profibrotic markers in TAC-induced renal fibrosis in rats. (**I, J, K**) are representatives of immunohistochemical staining of kidney tissues of different groups. (**A-D**) collagen type1, (**E-H**) α-SMA, and (**J, K**) their quantitative analysis. Data are represented as % of positively immunostained cells, results are representatives of at least 10 photographs for each group. Scale bars represent 50 μm. (**L**) Representative of TGF-β1 contents in kidney tissues determined by ELISA assay (*n* = 6). Results are expressed as mean ± SD; statistical significance was determined by one-way ANOVA followed by Tukey-Kramer multiple comparison test. ***a***: significantly different from the control group, ***b***: significantly different from the TAC group at *P* < 0.05

To confirm the histological findings, the expression of collagen type-1, and the pro-fibrotic marker α-SMA were determined by immunohistochemical staining. In addition, the renal content of TGF-β1 was identified by ELISA (Fig. 3). The TAC-treated group showed a marked cytoplasmic immunostaining reactivity for collagen type-1 in tubules (Fig. 3C, J), and a marked reactivity for α-SMA in glomeruli and tubules (Fig. 3G, K), with a statistically significant increase to 539%, and 360%, respectively. Moreover, the renal content of TGF-β1 increased to 854%, when compared to the control group (Fig. 3L). On the other hand, Lina co-treatment significantly decreased the expression levels of collagen type1 and α-SMA to 43%, and 42%, respectively, and the renal content of TGF-β1 to 65%, compared to the TAC-treated rats (Fig. 3D, H).

### Effect of Lina on TAC-induced oxidative stress in rat kidney tissues

**Fig. 4:**
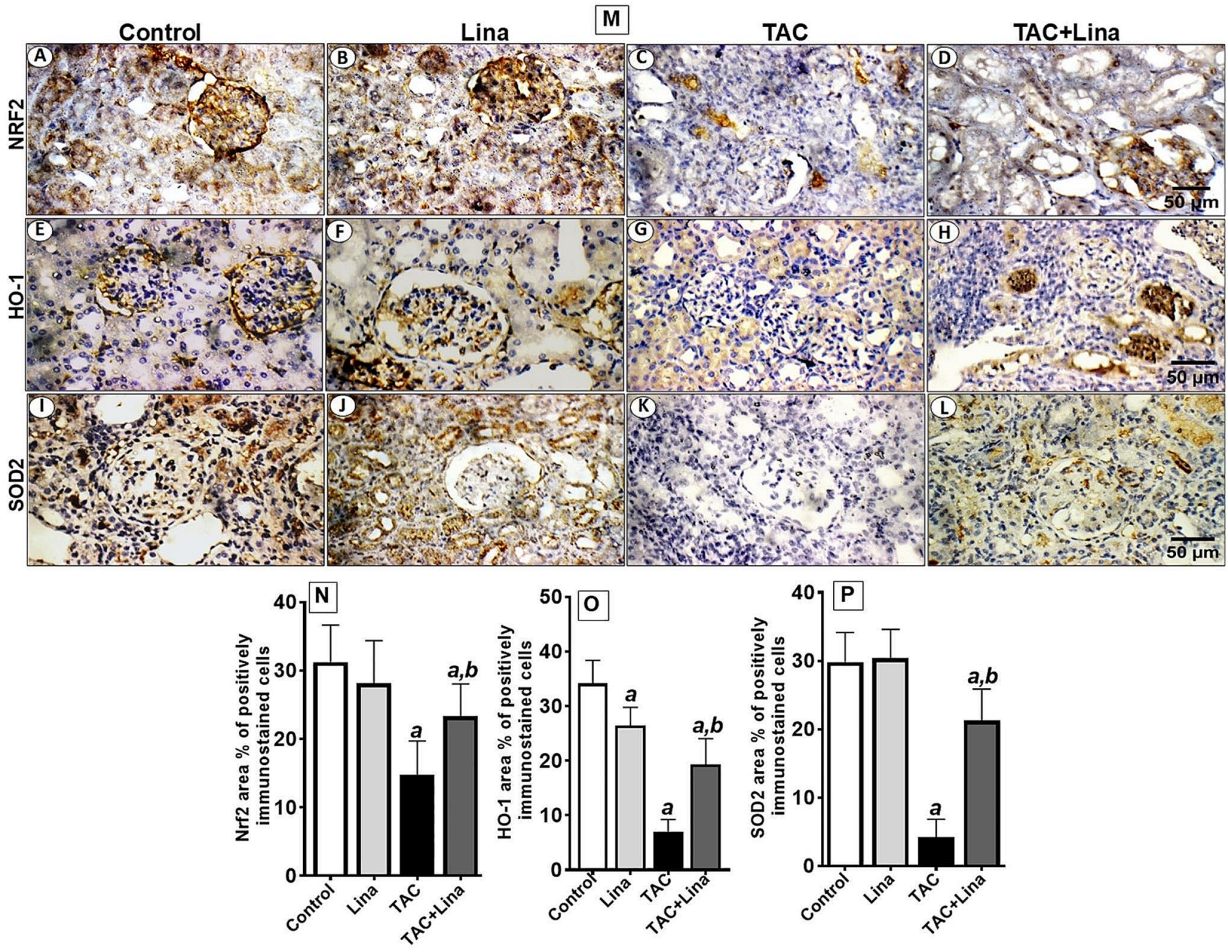
Effect of Lina treatment on the transcription factor Nrf2 and downstream antioxidants in TAC-induced oxidative stress in rats. (**M**) Representative immunohistochemical staining of kidney tissues. (**A-D**) Nrf2, (**E-H**) HO-1, (**I-L**) SOD2 and (**N, O, P**) the corresponding quantitative analysis. Original magnification 400x. (**A, E, I**) kidney sections of control and **(B, F,J)** kidney sections of Lina showing marked nuclear reactivity in glomeruli and tubules for Nrf2, marked cytoplasmic staining for HO-1 in glomeruli and tubules and marked cytoplasmic staining for SOD2 in tubules; (**C, G, K**) kidney sections of TAC showing moderate nuclear reactivity for Nrf2 in in tubules, moderate cytoplasmic reactivity for HO-1 in tubules and weak reactivity for SOD2 in tubules; (**D, H, L**) kidney sections of TAC + Lina showing moderate tubular reactivity for all markers in tubules. Data are represented as % of positively immunostained cells, results are expressed as mean ± SD, and are representatives of at least 10 photographs for each group. Scale bars represent 50 μm. Statistical significance was determined by one-way ANOVA followed by the Tukey-Kramer multiple comparison test. ***a***: significantly different from the control group, ***b***: significantly different from TAC group at *P* < 0.05

**Fig. 5:**
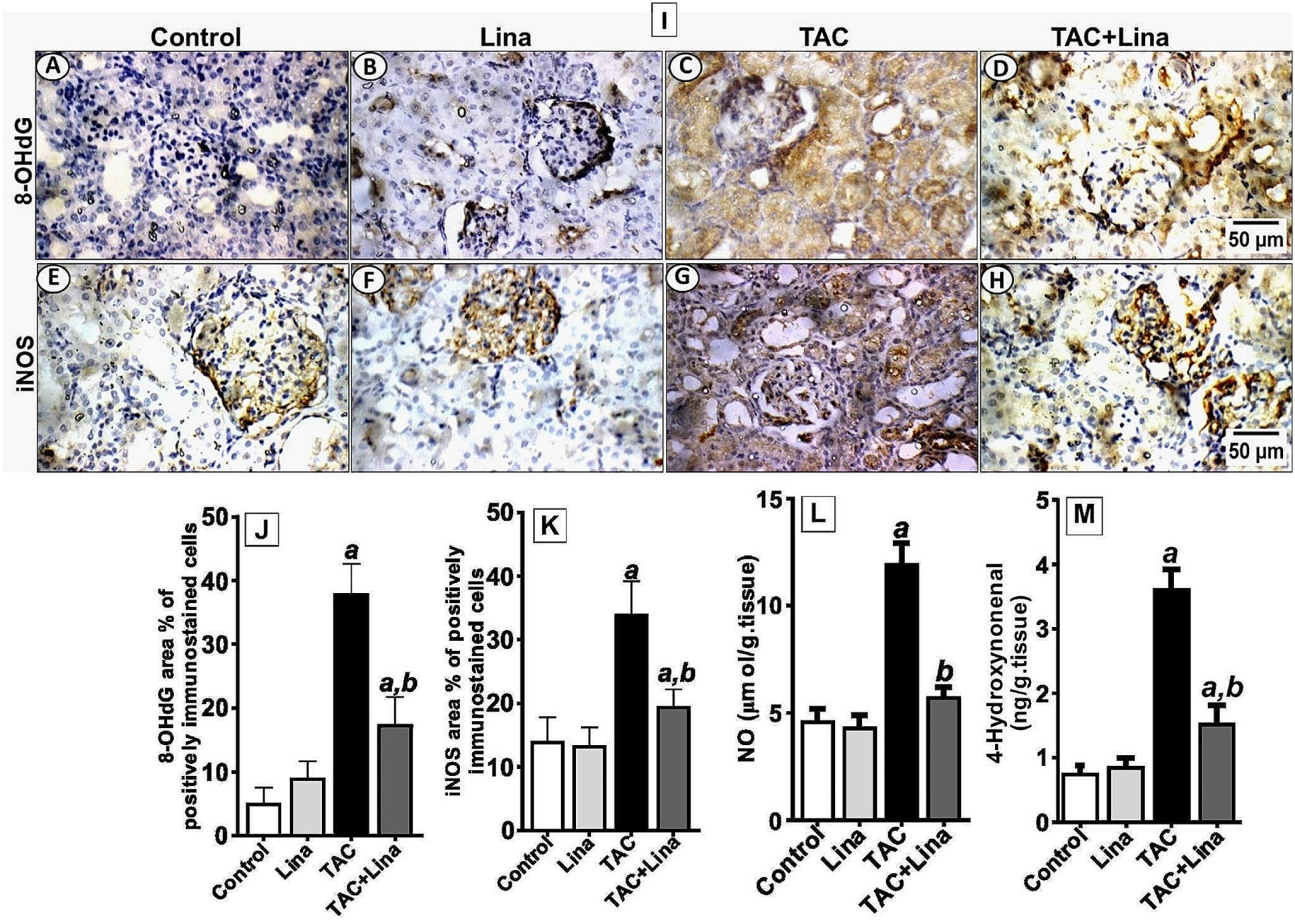
Effect of Lina treatment on oxidative stress biomarkers in TAC-induced renal injury in rats. (**I**) representatives of immunohistochemical staining of kidney tissues of different groups. (**A-D**) 8-OHdG, (**E-H**) iNOS, and (**J, K**) the corresponding quantitative analysis. Data are represented as % of positively immunostained cells, results are representatives of at least 10 photographs for each group. Scale bars represent 50 μm. (**L, M**) Representative colorimetric and ELISA assay of renal contents of NO and 4-Hydroxynonenal (*n* = 6). Results are expressed as mean ± SD, statistical significance was determined by one-way ANOVA followed by Tukey-Kramer multiple comparison test. ***a***: significantly different from the control group, ***b***: significantly different from the TAC group at *P* < 0.05

Tacrolimus caused a significant increase in the level of renal 4-hydroxynonenal, a sensitive marker of lipid peroxidation, and the immunostaining reactivity of 8-OHdG, an indicator for DNA oxidation (Fig. 4C), by 373%, and 616%, respectively, when compared to the control group (Fig. 4J and M). Besides, TAC administration caused a marked decrease in the protein expression level of SOD2 in kidney tissues to 86% (Fig. 5K, P). In contrast, administration of Lina disclosed a significant decrease in the renal content of 4-hydroxynonenal, and the area % of the immunostaining reactivity of 8-OHdG to 58%, and 51%, respectively. Also, it demonstrated a remarkable increase in protein expression of SOD2 to 411%, when compared to the TAC group (Figs. 5L and 4D and M).

### Effect of Lina on Nrf2 and HO-1 enzyme immunostaining reactivity in TAC-treated rats

The Nrf2 is a transcription factor that is necessary for regulating the expression of many antioxidant proteins that protect against oxidative damage. We investigated whether Lina activated the Nrf2 and the downstream heme oxygenase 1 enzyme. Our immunohistochemical assay showed that the protein expression level of Nrf2 was decreased by treatment with TAC. This is proved by a marked decrease in nuclear immunostaining reactivity in glomeruli, and in tubules to 56% (Fig. 5C, N). In comparison, the control groups showed remarkable nuclear reactivity for Nrf2 in glomeruli, and tubules (Fig. 5A). These events were intensely attenuated by co-administration with Lina that showed a moderate immunostaining reactivity in glomeruli, and tubules (Fig. 5D, N). Moreover, the protein expression level of HO-1 decreased to 79% with TAC treatment, as evidenced by negative cytoplasmic immunostaining reactivity (Fig. 5G, O). On the other hand, treatment with Lina significantly increased the area % expression of HO-1 to 189%, compared to the TAC group (Fig. 5H, O).

### Effect of Lina on NO content and immunostaining reactivity of iNOS in TAC-treated rats

As presented in Fig. 5, administration of TAC resulted in increasing total nitrite content in kidney tissues to 160%, and the protein expression level of iNOS to 130%, as evidenced by a marked cytoplasmic immunostaining reactivity in glomeruli and tubules, when compared with the control group (Fig. 4G, K, L). On the contrary, co-treatment with Lina significantly decreased the total nitrite content (Fig. 4H, L), and the area % of the expression of iNOS to 41%, in comparison to the TAC group.

### Effect of Lina administration on tissue levels of apoptotic markers

**Fig. 6:**
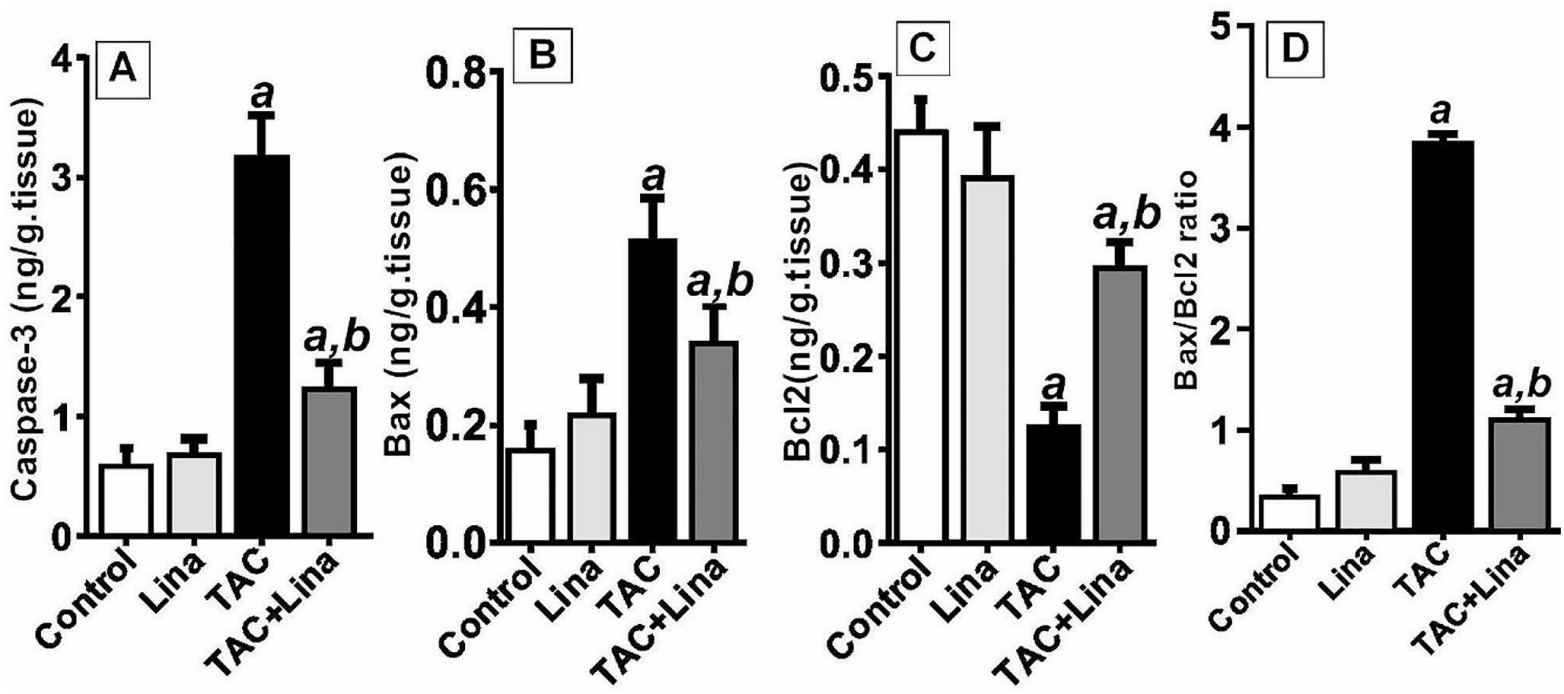
Effect of Lina treatment on apoptotic markers in TAC-induced renal injury in rats. (**A-D**) Representative ELISA assay of renal contents of caspase-3, Bax, Bcl2, and Bax/ Bcl2 ratio respectively. Data are presented as means ± SD (*n* = 6 per group) and statistically analyzed by a one-way parametric ANOVA test, followed by a post hoc Tukey-Kramer multiple comparison test. ***a***: significantly different from the control group, ***b***: significantly different from the TAC group at *P* < 0.05

Following TAC administration, the caspase-3, and Bax contents in kidney tissues markedly increased to 433%, and 225%, respectively. At the same time, the renal content of Bcl2 decreased to 71%, and the Bax/ Bcl2 ratio notably increased to 983%, when compared with the control group. In contrast, treatment with Lina caused a significant decline in renal contents of caspase-3, Bax, and Bax/ Bcl2 ratio to 62%, 35%, and 72%, respectively. Nevertheless, it led to a significant increase in Bcl2 renal content to 131%, when compared with the TAC group (Fig. 6).

### Effect of Lina on hypoxia and related profibrogenic proteins induced by TAC

**Fig. 7:**
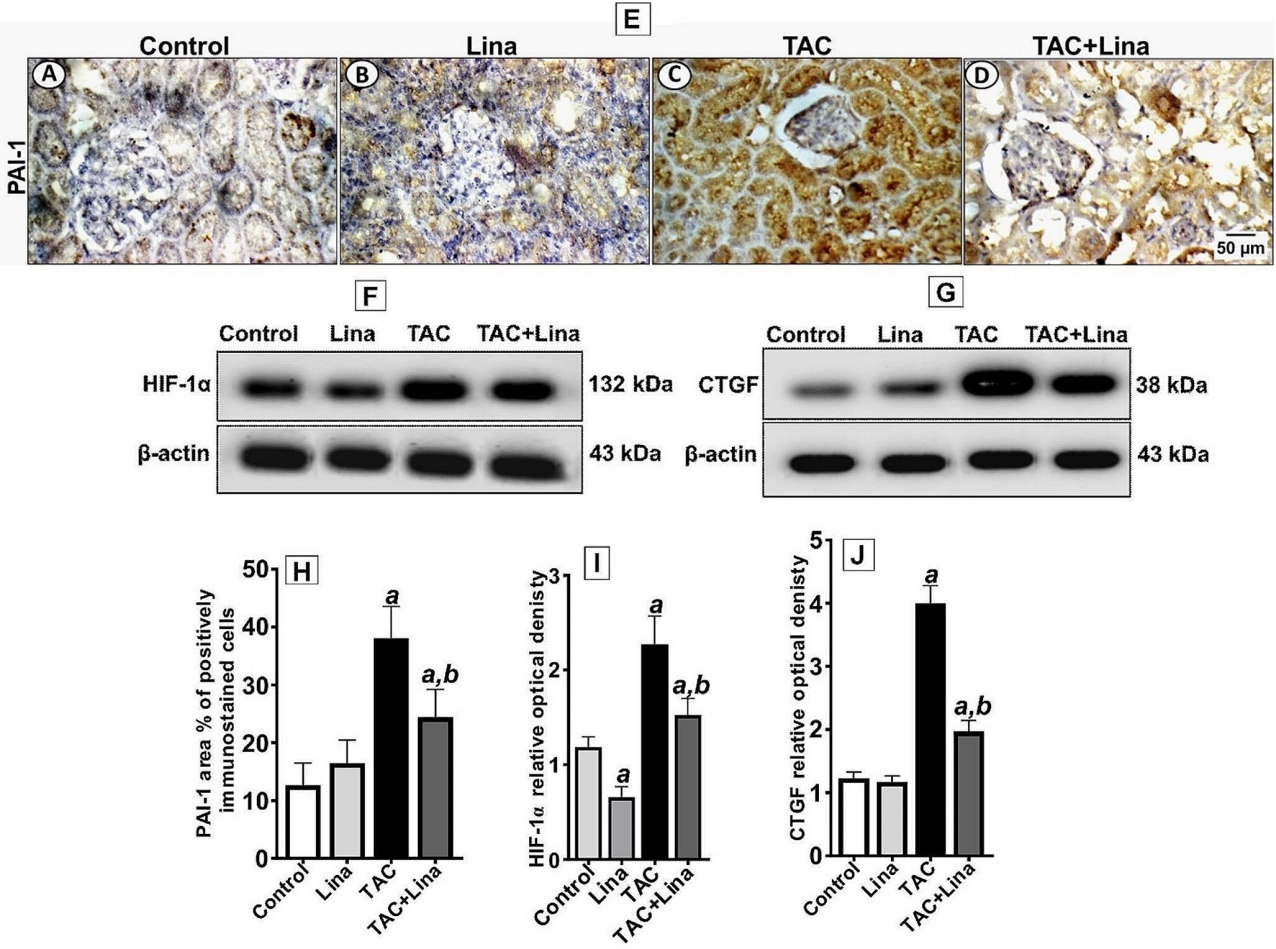
Effect of Lina administration on HIF-1α and profibrogenic proteins in TAC-induced hypoxia in rats. (**E**) Representative of immunohistochemical staining of PAI-1 and (**H**) the corresponding quantitative analysis. Data are represented as % of positively immunostained cells, results are representatives of at least 10 photographs for each group. Results are expressed as mean ± SD. Scale bars represent 50 μm. (**F, G**) Representative Western blots for HIF-1α and CTGF and (**I, J**) the corresponding quantitative analysis of the expression in kidney sections. Data were quantified by using Image Lab software and are represented as the relative optical density of bands to the β-actin band from the same gel. Results are expressed as mean ± SD of three individual experiments. Statistical significance was determined by one-way ANOVA followed by post hoc Tukey-Kramer multiple comparison test. ***a***: significantly different from the control group, ***b***: significantly different from the TAC group at *P* < 0.05

To investigate the effects of Lina on hypoxia, and its related signaling proteins in TAC-treated rats, we measured the protein expression of HIF-1α, CTGF, and PAI-1 in kidney tissues. Our results have revealed that the protein expression levels of HIF-1α, and CTGF increased in the renal tissues of the TAC group to 91%, and 235%, respectively, when compared to the control group (Fig. 7F, G). Moreover, our immunohistochemical examination of the kidney sections of TAC showed a marked cytoplasmic reactivity of PAI-1 (a fibrinolysis inhibitor that inhibits the degradation of the extracellular matrix, and matrix metalloproteinases) in tubules with a statistically significant increase in the area % of immunostaining reactivity to 188%, in comparison to the control group (Fig. 7E). Conversely, the Lina co-treatment significantly decreased the corresponding protein expression levels of HIF-1α, and CTGF to 35%, and 50%, respectively (Fig. 7I, J), as well as the area % of tubular expression of PAI-1 in kidney tissues to 37%, when compared to the TAC group (Fig. 7H).

## Discussion

Although serious side effects hinder its long-term administration, tacrolimus remains the best immunosuppressant medication for preventing graft rejection after transplantation, and for a wide range of autoimmune diseases [5, 25]. It has been documented that tacrolimus induces a permanent nephrotoxicity identified through vascular resistance, ischemia, hypoxia, apoptosis, and interstitial fibrosis that ultimately contribute to chronic kidney dysfunction [4, 6, 26].

The present study has been carried out to explore the potential protective role of Lina in a rat model of TAC-induced renal injury and to elucidate the underlying molecular mechanisms.

Our results have proved that Lina exhibited anti-oxidant and antiapoptotic properties. Furthermore, Lina treatment modulated hypoxia, and its associated target proteins which contribute to fibrosis progression.

In the current study, treatment with TAC for 4 weeks showed a significant increase in S_cr_ and BUN/ S_cr_ ratio as well as urinary cystatin C levels accompanied by a significant decrease in Cl_Cr_ and serum albumin compared with the control group, indicating kidney dysfunction. Our findings are similar to what has been previously reported [6, 27].

Lina’s renoprotective efficacy was confirmed by improved kidney dysfunction as demonstrated by improvements in the aforementioned renal biomarkers. These results are consistent with those of previous studies [12, 28].

Interstitial collagen deposition, striped fibrosis, tubular atrophy, and thickened glomeruli are the main histopathological alterations considered specific for TAC nephrotoxicity. This has been confirmed in our results and is consistent with previously reported literature [29].

Furthermore, TGFβ-1, a profibrotic cytokine, is a critical regulator of kidney fibrosis, and in the production and degradation of ECM. TGFβ-1 can induce fibroblast differentiation into myofibroblasts, which are identified by α-SMA expression. Myofibroblasts then produce ECM proteins where excess deposition of these proteins can harm normal kidney structure, and interfere with renal function [30]. Our results have revealed that the renal content of TGFβ-1, as well as the protein expression of α-SMA and collagen type1, increased following TAC treatment. These align with the results published by Lim et al. [1], where the immunostaining of fibrotic markers collagen, fibronectin, and α-SMA increased in the TAC group. In addition, the study of Ume and colleagues [31] showed that tacrolimus-treated renal fibroblasts displayed fibroblast-to-myofibroblast transition phenotyping, which was demonstrated by α-smooth muscle actin, and collagen type IV overexpression accompanied by concomitant induction of TGF-β1 ligand secretion, and receptor activation. It has been reported that DPP-4 is important for TGF-β receptor hetero-dimerization and signaling in human microvascular endothelial cells [32]. Moreover, the DPP-4 has been recognized as a marker of a highly activated subset of myofibroblasts, and its inhibition results in lowered ECM deposition in cultured fibroblasts [33]. Furthermore, DPP-4 has been confirmed to bind to collagen type 1 and fibronectin through its cysteine-rich domain located at the extracellular portion of the enzyme. This promotes cell migration and forms the structure of the fibrotic microenvironment [11]. In the current study, Lina as a selective DPP-4 inhibitor significantly decreased the tubular injury score and the % of the fibrotic area within the kidney interstitium. Moreover, our immunohistochemical results have shown a lower % of immunostaining for α-SMA and collagen type1, and a decreased content of TGF-β1 in kidney tissues treated with Lina. These results together with the attenuation of kidney function biomarkers suggest that Lina effectively mitigated the progression of fibrosis in our model. Our findings also agree with those of a previous study by Bai et al. [34], where Lina downregulated the expression levels of profibrotic proteins, and attenuated the histopathological deterioration in a rat model of angiotensin II-induced kidney fibrosis.

One of the primary mechanisms involved in TAC-induced kidney injury is an alteration in redox homeostasis [4, 5]. It is acclaimed that oxidative stress is mainly caused by an imbalance between ROS and cellular anti-oxidative defensive mechanisms [35]. Obviously, nitric oxide may react with the superoxide radical to produce peroxynitrite, an RNS; and the resulting nitrosative stress can cause further kidney tissue damage [36]. Additionally, ROS or RNS can react with intracellular lipids, nucleic acids, and proteins, leading to lipid peroxidation, DNA damage, and protein denaturation, subsequently apoptotic cell death, and damage to tissue structure and function [9, 37]. Administration of TAC in the current study significantly increased the levels of oxidative stress biomarkers including 4-hydroxynonenal and 8-OHdG in kidney tissue, whereas the expression level of the antioxidant enzyme SOD2 has been decreased, compared with that of the control group. Our observations are similar to Gao and colleagues’ findings [5] showing that TAC causes oxidative stress in mice by increasing MDA levels, and lowering the activity of antioxidant enzymes superoxide dismutase, catalase, and glutathione peroxidase in kidney tissue. Furthermore, the study conducted by Kim et al. [38] showed that TAC administration increased the levels of 8-OHdG in urine and kidney tissue, and the renal content of 4-hydroxynonenal. Our results have also revealed that there was a significant increase in the renal content of total nitrites, which represent the endogenous nitric oxide content, and an intensive immunostaining reactivity of iNOS in TAC-treated rats, compared to control rats. Similar effects have been reported in the study of Back et al. [7]. On the other hand, our results have demonstrated that Lina co-treatment increased the expression level of SOD2, and decreased the protein expression of 8-OHdG, iNOS, and renal contents of 4-hydroxynonenal, and NO in kidney tissues. The antioxidant effects of Lina could be ascribed to its unique chemical structure among other DPP-4 inhibitors since it has a xanthine backbone and can inhibit the xanthine oxidase activity which plays an important role in purine metabolism and cellular oxidative status. In addition, previous studies showed that Lina could inhibit NADPH oxidase enzyme thus decreasing the generation of ROS [39]. These protective effects of Lina against oxidative stress are parallel with those mentioned by Arab et al. [16], and Wu et al. [13].

Nrf2 is an essential transcription activator for cellular redox homeostasis. It maintains the balance between ROS and free radical scavenging enzymes and antioxidants. Once activated by oxidative stress, Nrf2 was uncoupled from Kelch ECH-associating protein 1 (Keap1) Then, it was translocated to the nucleus to bind with the antioxidant response element (ARE) of the target genes to promote the transcription of these antioxidant genes [5]. It is known that Nrf2 controls the transcription of over 300 ARE-regulated genes [40]. The immunostaining of Nrf2 and the assessment of its cellular localization (cytosolic versus nuclear) is a reliable indicator of translocation and transcription activation. Furthermore, the assessment of the expression of downstream antioxidant genes, including HO-1, and SOD, is another reassurance finding indicating transcription activation of Nrf2.

In the present study, the TAC group has shown a marked decrease in the nuclear immunostaining of Nrf2, and a significant decrease in the cytoplasmic immunostaining of the downstream HO-1 enzyme, when compared with the control groups that has reflected intense nuclear and cytoplasmic staining for Nrf2, and HO-1, respectively. Moreover, our IHC results have revealed that there is a parallel glomerular, and tubular expression pattern of Nrf2 and its target downstream proteins with nearly equivalent immunostaining reactivity, which might suggest a functional relationship. Our findings confirm the results reported by Azouz et al. [41], Ibrahim et al. [42], and Gao et al. [5]. Oppositely, the administration of Lina significantly increased the protein expression level of Nrf2 and HO-1 in kidney tissues of TAC-treated rats, as evidenced by an increase of the area % of the immunostaining of the corresponding proteins. Our findings are in line with that of Wu and colleagues [13] who stated that Lina ameliorated endotoxin-shock-induced acute kidney injury in rats and cell line models through upregulation of Nrf2/HO-1 protein expression. A parallel effect was previously documented by the study of Mima et al. [43], where Lina has offered protection against diabetic kidney injury partially *via* activating the Keap1/Nrf2 pathway on the high glucose-induced podocyte apoptosis model. Furthermore, Spencer and colleagues [44] described that Lina ameliorated albuminuria and kidney hypertrophy in diabetic DBA/2J mice, and increased the mRNA and protein levels of the antioxidants CAT and MnSOD in glucose-6-phosphate dehydrogenase deficient mice.

HIF-1α is the major controller of the cellular adaptation to hypoxia. In addition, it contributes to the initiation and development of kidney fibrosis [8]. As a transcription factor, HIF-1α activation upregulates the expression of profibrotic genes and biosynthetic enzymes [45]. Moreover, a direct relative association between NO, iNOS, and HIF-1α exists. When NO is produced in abundance, it upregulates HIF-1α under hypoxic conditions. Besides, HIF-1α increases the iNOS gene expression [35]. Furthermore, it has been postulated that oxidative stress modulates hypoxic factors whereby, during hypoxia, mitochondria increase the production of ROS, which inhibits prolyl hydroxylase domain enzyme activity, and consequently stabilizes the HIF-1α protein [9]. In the present study, our data have displayed that the expression level of HIF-1α significantly increased after TAC treatment. Besides, the expression levels of the profibrotic proteins, which were mediated by HIF-1α, including CTGF and PAI-1 increased as well. It is noteworthy that our research is the first in the literature to demonstrate the direct significant role of HIF-1α in the TAC-induced renal injury model. Actually, a few studies have reported the role of the profibrotic proteins on TAC nephropathy. The study by Knops et al. [46] indicated that the expression of CTGF increased in proximal tubular cells following TAC exposure. On the contrary, in Lina co-treatment, the protein levels of HIF-1α, and CTGF significantly decreased; and PAI-1 showed a notable decrease in immunostaining reactivity in kidney tissues. Therefore, it has been reported that Lina protects the microvascular endothelial cells from hypoxia by modulating the SIRT1/HIF-1α/VEGF pathway [47]. In addition, Ishibashi and coworkers [48] stated that Lina inhibited the advanced glycation end-products-induced ROS in endothelial cells through the downregulation of the PAI-1 gene expression.

Apoptosis is a process of programmed cell death that plays a significant role in TAC-induced renal injury [4, 38]. It has been documented that oxidative stress is a common mechanism leading to apoptotic cell death. Jin and collaborators [49] previously mentioned that TAC-induced oxidative stress induces apoptotic and autophagic cell death, and that was closely associated with structural and functional kidney injury. Caspase-3 is known to be a key apoptotic mediator due to its catalytic activity on various critical cellular proteins. Bcl-2 is a protein that blocks programmed cell death by controlling mitochondrial apoptosis while The Bax protein, a member of the Bcl-2 family, promotes apoptosis. The ratio of Bax/ Bcl-2 is an indicator of the susceptibility of a cell to undergo apoptosis [1, 35]. In our results, TAC increased the renal contents of caspase-3 and Bax proteins and decreased that of Bcl-2. On the other hand, the Lina treatment modulated such an effect induced by TAC. Our results are in agreement with the findings by Korbut et al. [50] stating that Bcl-2 level increased, and caspase-3 expression decreased in Lina treated group in a model of diabetic kidney disease.

## Conclusion

In summary, linagliptin has attenuated TAC-induced renal injury in the experimental rat model used in this study through antioxidant, anti-hypoxic, and anti-apoptotic effects. The underlying mechanisms include modulation of Nrf2/HO-1 and HIF-1α/CTGF/PAI-1 axis.

## Electronic supplementary material

Below is the link to the electronic supplementary material.

Supplementary Material 1

## Data Availability

No datasets were generated or analysed during the current study.
