# Supplementary material for: Linagliptin ameliorates tacrolimus-induced renal injury: role of Nrf2/HO-1 and HIF-1α/CTGF/PAI-1
Source: Mol Biol Rep. 2024 May 5;51(1):608. doi: 10.1007/s11033-024-09533-2 (PMC11070395; doi:10.1007/s11033-024-09533-2)
Supplement: Supplementary file 1 — Supplementary Material 1 [file 11033_2024_9533_MOESM1_ESM.docx]

**Supplemental figures**

**Original western blot photos**

All the photos have been adjusted for the same brightness and contrast

**Fig. 7B:** **Representative Western blots for HIF-1α**

**Fig. 7C:** **Representative Western blots for CTGF**
